# Effect of 12-week unstable surfaces resistance training on muscle stiffness, muscle co-activation, and balance in older individuals

**DOI:** 10.1186/s11556-026-00430-0

**Published:** 2026-07-28

**Authors:** Ping Liu, Lele Chang, Yueyue Shi, Yaru Hao

**Affiliations:** 1 University of Malaya, Kuala Lumpur, Malaysia; 2 Jeonbuk National University, Jeonju, Korea, Republic of; 3 Changsha Normal University, Changsha, China

**Keywords:** Older adults, Unstable resistance training, Stable resistance training, Co-activation, Muscle–tendon stiffness, Balance

## Abstract

**Objective:**

This randomized controlled trial evaluated the effects of unstable resistance training (URT) and stable resistance training (SRT) on muscle stiffness, co-activation, balance, and pain in older adults. To date, no study has investigated dose-matched instability-based resistance training in this population with prespecified endpoints of co-activation and muscle stiffness.

**Methods:**

Forty-six older adults were randomly assigned to either the URT (*n* = 23) or SRT (*n* = 23) group for a 12-week intervention. Both groups performed seven lower-limb exercises with matched volume and intensity, with the URT group utilizing instability devices. Outcomes included muscle–tendon stiffness in the quadriceps and hamstrings, co-activation during squat-down (SD), stand-up (SU), and transition (SUSD). Balance was assessed using the Y-Balance Test (YBT) and Balance Error Scoring System (BESS), and pain was measured with the visual analogue scale (VAS).

**Results:**

Compared with SRT, URT resulted in greater reductions in co-activation during SD (*p* = 0.035), SU (*p* = 0.003), and SUSD (*p* = 0.001). URT also produced larger improvements in balance, as indicated by BESS (*p* = 0.009), YBT-right (*p* = 0.008), and YBT-left (*p* < 0.001). Increases in muscle stiffness with URT were limited to the vastus medialis, quadriceps tendon, and hamstring tendon (all *p* < 0.05), whereas SRT produced broader increases in muscle–tendon stiffness (all *p* < 0.05). VAS scores decreased in both groups, with a greater reduction after URT (*p* = 0.009).

**Conclusion:**

In older adults, URT more effectively reduced co-activation and enhanced balance under matched loads, while SRT increased muscle stiffness across a broader range of muscles. Given that both interventions reduced pain, the choice of intervention should depend on whether the primary objective is to improve coordination and balance or to increase muscle stiffness.

**Trial registration:**

UMIN Clinical Trials Registry, retrospectively registered. Registration number XXXX.

**Supplementary Information:**

The online version contains supplementary material available at https://doi.org/10.1186/s11556-026-00430-0.

## Introduction

Joint degeneration is a growing public health concern among adults over 60, affecting more than 10% of this population and rising with age [1, 2]. The knee is especially vulnerable, as older adults often experience decreased peri-knee muscle stiffness, higher co-activation, and impaired balance, all of which contribute to functional decline and diminished quality of life [3–5].

Older adults exhibit less quadriceps and hamstring stiffness than younger adults, due to smaller muscle area and greater intramuscular fat [6]. Muscle atrophy and fatty infiltration drive these changes and appear to underlie the decline in stiffness. Lower stiffness also correlates with decreased joint range of motion, suggesting age-related declines in stiffness may constrain movement amplitude and limit functional capacity [7–9]. Over time, these changes in muscle properties affect neuromuscular control and lead to compensatory co-activation [10].

Co-activation, the simultaneous contraction of agonist and antagonist muscle groups, is an important neuromuscular strategy for joint control and balance in young people [11]. Older individuals show approximately 18%–22% higher muscle co-activation levels than younger adults [12]. While initial co-activation stabilizes the joint and facilitates balance, excessive co-activation increases stiffness in periarticular soft tissues and the joint [13], thereby impairing task-specific postural adjustments [14, 15]. One proposed mechanism is age-related degradation of proprioception and sensorimotor control, which funnels neuromuscular output into fewer stabilizing pathways, promoting inefficient co-contraction [16, 17].

Despite extensive clinical trials on balance in older adults, muscle stiffness and co-activation have rarely been evaluated as rehabilitation outcomes in older adults. Conventional resistance training (RT) has been demonstrated to increase muscle stiffness and may reduce co-activation, thereby improving movement efficiency in young individuals [18]. However, achieving meaningful benefits may require higher loads or frequencies, which increases musculoskeletal risk in older adults. Moreover, its effects on balance in older adults have been inconsistent. Several studies report modest or negligible balance gains with RT alone [19, 20]. A systematic review found that only 22% of progressive RT programs significantly improved balance [21].

Against this backdrop, unstable resistance training (URT) has emerged as a practical modality that combines balance demands with external loading, challenging both muscular strength and the neuromuscular system [22]. By embedding proprioceptive and sensorimotor perturbations within functional strengthening, URT directly targets intermuscular coordination and may yield greater gains in balance than conventional resistance training [22]. However, clinical evidence for older adults remains limited, particularly in trials that concurrently evaluate lower-limb muscle stiffness and co-activation. Preliminary evidence indicates that URT increases motor-unit recruitment in peri-knee muscles and improves neuromuscular control, which may reduce lower-limb stiffness and maladaptive tension [23, 24]. By enhancing intermuscular coordination and peri-knee strength, URT may also limit compensatory co-activation [25, 26]. These promising signals motivate a randomized trial comparing URT and stable resistance training (SRT) in older adults, focusing on muscle stiffness, co-activation, balance, and pain. We hypothesize that URT, compared to SRT, will result in greater improvements in these outcomes among older adults. The findings may help inform the design of exercise interventions targeting neuromuscular control and balance performance, which are relevant to fall-related mechanisms in older adults.

## Materials and methods

### Participants

A priori sample size estimation was performed using G*Power 3.1.7 for ANCOVA, based on the primary outcome of muscle stiffness. The expected effect size was informed by a previous shear-wave elastography study that assessed quadriceps muscle shear modulus [27]. Using an effect size of f = 0.30, α = 0.05, power = 0.95, and two groups, the minimum required sample size was 40 participants. To account for an anticipated 20% dropout rate, 50 participants were recruited.

The inclusion criteria were: (a) Aged 65 to 75 years old; (b) Be able to walk independently on level ground, up and down stairs, with normal joint activities (Flexion and Extension); (c) No history of hip, knee or ankle pain, surgery or trauma in the last six months; (d) Voluntarily participate in the test and sign an informed consent form. The exclusion criteria were: (a) Suffer any neuromusculoskeletal severe injuries or diseases; (b) Suffer serious cardiovascular and cerebrovascular diseases and mental disorders; (c) Unable to cooperate with the actions required to complete the study; (d) History of allergic to surface electrodes; (e) Suffer other medical severe diseases or other reasons resulting to inability to complete the test.

The screening process for healthy older adults was conducted before participant enrollment during the baseline assessment phase (January to March 2024). Trained physiotherapists conducted the screening. Participants were recruited via community advertisements, referrals, and recommendations, providing written consent. The study was conducted under the principles of the Declaration of Helsinki and approved by the Ethics and Research Committee of XXX University (No.2024- 061). All participants signed a written informed consent before baseline assessments.

**Fig. 1:**
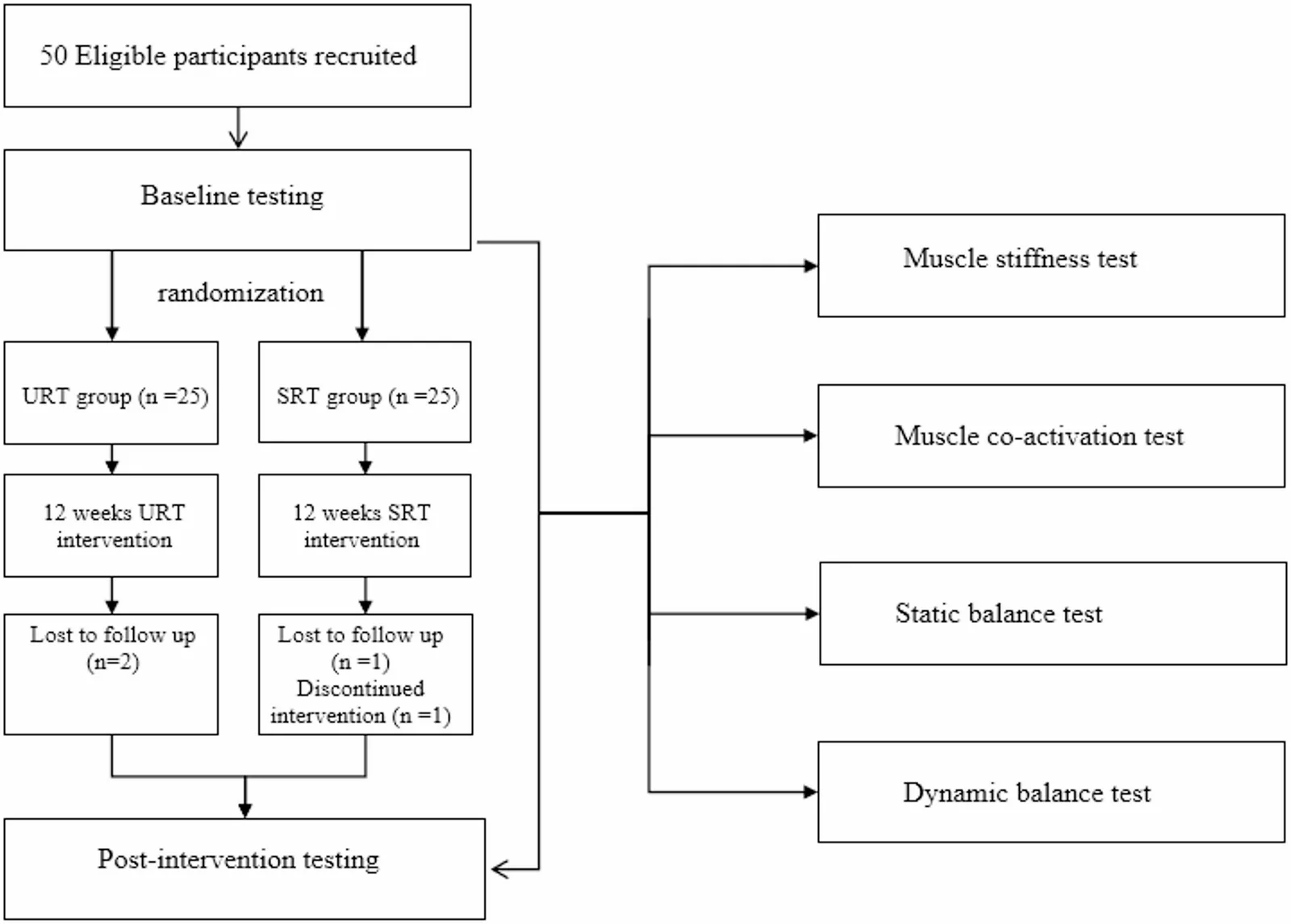
Participants allocation and experimental design

An independent operator generated a 1:1 computer-based random allocation sequence before group assignment. After completion of baseline assessments, 50 participants were allocated to either the URT group (*n* = 25) or the SRT group (*n* = 25) according to this sequence. Allocation concealment was maintained using sequentially numbered, opaque, sealed envelopes that were opened only after baseline testing was completed. The operator was not involved in outcome assessments or in the delivery of interventions. Assessments occurred at baseline and week 12 (Fig. 1).

### Interventions

#### Developing and designing the intervention program

The intervention was developed with expert input and aligned with prior work [23, 28]. Nine certified trainers prepared standardized, visually guided materials to ensure protocol consistency across sessions and sites (Table 1). Participants continued their usual pain-management regimen throughout the intervention.

**Table 1:** Rehabilitation protocol group with URT

Component	URT group	SRT group
Surface / Support	Unstable, non-fixed platform; progressively higher-instability levels with unpredictable perturbations.	Stable platform or firm ground.
Footwear	Barefoot	Barefoot
Sets × Reps	3 sets × 10–15 reps per exercise	3 sets × 10–15 reps per exercise
Intensity (prescription)	Borg RPE 6–7	Borg RPE 6–7
Rest between sets	60–90 s	60–90 s
Rest between exercises	2–3 min	2–3 min
Progression rule	Advance to a higher-instability platform (greater perturbation/instability level) when the upper rep limit is reached for 2 consecutive sessions	Increase load by 5–10% at the next session when the upper rep limit is reached for 2 consecutive sessions
Volume monitoring	Record sets, reps, load; total volume = sets × reps × resistance	Record sets, reps, load; total volume = sets × reps × resistance

- SRT training program

Participants in the SRT group engaged in a 12-week intervention consisting of three 60-minute sessions per week (10-minute warm-up, 40-minute training, and 10-minute cool-down). Each session consists of seven exercises targeting major muscle groups, as outlined by Eckardt [23]. The program includes seven lower-limb movements: squat, forward lunge, lateral lunge, wall sit (isometric), single-leg Romanian deadlift, glute bridge, and standing hip extension/kickback. These exercises use free weights, machines (Physicus, Auriflama - SP, Brazil), and elastic bands (Thera-Band, Hygenic Corporation, Akron, Ohio) for resistance.

Each session began with a brief demonstration to standardize setup and standardized verbal cues (e.g., “maintain a neutral pelvis” and “align knee with the second toe”). Participants then trained barefoot on a stable platform or firm ground under direct supervision, with real-time feedback on alignment. Movement tempo was standardised to an approximate 1:2 concentric-to-eccentric ratio. Rest intervals were standardised to 60–90 s between sets and 2–3 min between exercises.

Training intensity was guided using the Borg Rating of Perceived Exertion (RPE) scale, with participants selecting a load that corresponded to an RPE of 6–7 [29, 30]. Each exercise involved three sets of 10–15 repetitions, with the external load adjusted as needed to maintain the target RPE range across the prescribed repetition span. If a participant exceeded the upper limit of the range for two consecutive sessions, the resistance was increased by 5–10% in the subsequent session.

(b) URT training program

The URT group performed the same exercises and dosage as the SRT group, with the addition of instability devices: a BOSU^®^ ball (BOSU^®^, Ashland, OH, USA), a balance disc (AIREX AG, Sins, Switzerland), and a physioball (TheraBand^®^, Performance Health/Hygenic Corporation, Akron, OH, USA). All sessions took place on an unstable, non-fixed platform equipped with a waist-height rail for light fingertip support.

Each session began with a 10-minute familiarization period, followed by a no-load adaptation bout on the platform. Participants then performed the same seven lower-limb exercises as the SRT group. Dosage, footwear, cues, and rest intervals matched the SRT protocol. Rail contact was minimized and recorded.

Instability progression followed a predetermined sequence, with each device matched to its respective challenge level. For standing tasks, the sequence was firm ground, balance disc, BOSU^®^ dome-up, and BOSU^®^ dome-down. For supine tasks, such as the glute bridge or hamstring curl, the order was floor, feet on physioball (bilateral), and feet on physioball (single leg). Participants progressed to the next instability level after achieving the maximum number of repetitions in two consecutive sessions while maintaining correct form and minimal rail contact. If these criteria were not met, participants remained at the current instability level.

(c) Adverse events monitoring

Trained assessors supervised all sessions, provided feedback, and corrected unsafe or poorly aligned movements. Participants who reported knee pain or stiffness during exercise received lower resistance loads and reduced intensity. Monthly face-to-face or telephone interviews monitored adverse events by systematically inquiring about health deterioration or new musculoskeletal and cardiometabolic issues related to the intervention.

(d) Blinding of training allocation

All participants were required to perform either the URT or SRT exercises. Because the two exercise programs differed, blinding the participants was not feasible. To reduce assessment bias, outcome assessors were blinded to group allocation throughout data collection.

### Outcome measures

The primary outcome was muscle stiffness assessed by shear-wave elastography (SWE). Secondary outcomes included muscle co-activation, static and dynamic balance performance, and pain intensity. All outcomes were assessed at baseline (before randomization) and again after the 12-week intervention, approximately 48 h after the final training session. Assessments were conducted by two trained physiotherapists, with good-to-excellent measurement reliability (ICC > 0.85).

#### Demographics and pain

At baseline, age, gender, educational level, marital status, socioeconomic status, and employment were recorded from all eligible participants. Body mass (kg) and height (m) were measured using a mechanical scale and stadiometer (SECA, Germany). Participants were barefoot and wore light clothing. The measurements followed the guidelines of the International Society for the Advancement of Kinanthropometry (ISAK) [31]. Pain intensity was assessed using the Visual Analogue Scale (VAS), a 10 cm line ranging from 0 (no pain) to 10 (worst pain imaginable) [32].

#### Muscle stiffness

Muscle stiffness was assessed in the quadriceps and hamstrings using an ACUSON Oxana2 ultrasonic diagnostic system with VTIQ mode and a 9L4 linear transducer (7–9 MHz). Participants were positioned supine for quadriceps measurements and prone for hamstring measurements, keeping the tested limb fully relaxed throughout. For quadriceps, the rectus femoris (RF), vastus medialis (VM), and vastus lateralis (VL) were obtained at the midpoint of the line from the anterior superior iliac spine (ASIS) to the superior patellar border, directly on each muscle belly. Subsequently, quadriceps tendon stiffness was measured at the superior patellar border (midline). For hamstrings, the biceps femoris (BF) and semimembranosus (SM) were measured at the midpoint of the line between the ischial tuberosity and popliteal fossa. Hamstring tendon stiffness was measured at the inferior margin of the ischial tuberosity [33, 34].

The probe was aligned parallel to muscle fibers. After stabilizing the elastography sampling box (square imaging field) for approximately 5 s, the image was frozen, and shear wave velocity (SWV) was measured using a 1 × 1 mm region of interest (ROI) placed within the box. Three images with three ROIs per muscle were obtained, and mean values were analyzed. Measurements were obtained from the dominant limb for standardization across participants. All measurements were performed by the same examiner. Further technical details are provided in the Supplementary Appendix 1.

#### Muscle co-activation

Muscle activity of the RF, VL, VM, BF, and semitendinosus (ST) was recorded using surface EMG (FreeEMG1000, BTS Bioengineering, Milan, Italy). Surface landmarks for these muscles (RF, VL, VM, ST, and BF) were identified manually by a single trained examiner following established guidelines [35, 36]. To ensure reliability, all electrode placements were verified using manual muscle testing and rechecked for consistency during repeated trials. Skin preparation included shaving excess hair and cleaning with NuPrep Skin Gel before applying surface electrodes.

All participants performed maximal voluntary isometric contractions (MVICs) to normalize EMG data during squat trials (Supplementary Appendix 2). Subsequently, all participants performed five squat trials (feet shoulder-width apart), comprising squat-down (SD), stand-up (SU), and the transition (SUSD) phases.

The time-varying multi-muscle co-activation function (TMCf) was used to evaluate co-activation between quadriceps and hamstring muscle groups across the movement phases [37]. Participants performed SD, SU, and SUSD tasks with their feet at shoulder width and their second toes pointing forward while maintaining an upright back posture (Supplementary Appendix 3). Each movement was measured five times at 5-minute intervals. EMG data analysis determined the average root mean square (RMS) value across three measurement periods [36].

#### Balance Assessment

##### Dynamic Balance

Assessed using the Y-Balance Test (YBT), a modified version of the Star Excursion Balance Test [38, 39]. Participants balanced on one foot, aligned their big toe with a starting line, and pushed a test board to reach as far forward, backwards, and sideways as possible. Distances were recorded with 0.5 cm accuracy across three repetitions per direction, alternating feet and repeating. After 4–6 familiarization practice attempts to ensure stable performance, participants completed three actual reach trials in each direction. Absolute reach distances from these trials were then normalized to leg length, and the normalized values were used to compute the YBT composite score [40]. Leg length was measured from the anterior superior iliac spine to the most prominent bony point of the ipsilateral medial malleolus with a standard tape measure while participants lay supine on a bed. The final normalized composite reach distance was calculated for each leg as the sum of the maximum reach distance (cm) in all three directions, divided by 3 times the limb length (cm) and multiplied by 100 [41].

##### Static Balance

Assessed using the Balance Error Scoring System (BESS), a commonly used and reliable tool for assessing balance in clinical and sport-related populations [42, 43]. The BESS comprises six 20s trials, including double-leg, single-leg, and tandem stances performed on both firm and foam surfaces, with participants standing barefoot, eyes closed, and hands placed on the iliac crests [42, 43]. During each trial, errors such as opening the eyes, lifting the hands from the iliac crest, stepping, stumbling, or moving the feet from the test position were recorded, with each error scored as 1 point (maximum 10 per trial; total score 0–60). Each trial was separated by a rest period of 20 to 30 s. Before testing, participants completed one familiarization trial per surface. A trained assessor, blinded to group allocation, recorded errors in real time. The total score was the sum of all errors, with higher scores indicating poorer balance performance.

### Statistical analysis

Statistical analyses were conducted using SPSS software, Version 24.0 (IBM Corp., Armonk, NY, USA). Data normality and homogeneity were evaluated, and ANCOVA assumptions were checked before the corresponding analyses. Measurement indicators are presented as mean (M) and standard deviation (SD). Baseline comparisons of clinical outcomes were performed using independent-samples t-tests (for parametric data) or Mann–Whitney U tests (for non-parametric data). The paired-samples t-test (for parametric data) and the Wilcoxon signed-rank test (for nonparametric data) were used to analyze differences between baseline and week 12 measurements within each group. Between-group effects were prioritized. Outcomes meeting ANCOVA assumptions were analyzed using baseline-adjusted ANCOVA; otherwise, change scores were compared between groups using Mann–Whitney U tests. Holm adjustment was applied for multiple comparisons. Effect sizes were reported as ηp² for ANCOVA and r for Mann–Whitney U tests. Statistical significance was set at *p* < 0.05.

## Results

### Characteristics of the overall sample (*N* = 50)

A total of 70 older adults were screened. After accounting for screening and refusals, 50 participants were enrolled and randomized to either the URT group (*n* = 25) or the SRT group (*n* = 25) (Fig. 1). The specific reasons for exclusion are shown in Fig. 1. Baseline demographics and clinical measures did not differ between groups (Table 2).

**Table 2:** Baseline characteristics of participants

Outcome measures	URT	SRT	*p*-Value
Age (years)	70 (3)	68.7 (2.29)	0.186
Gender, n (%)			0.747
Male	19 (76.0)	18 (72.0)	
Female	6 (24.0)	7 (28.0)	
Marital status, n (%)			0.185
Married	17 (68.0)	21 (84.0)	
Unmarried/other	8 (32.0)	4 (16.0)	
Weight(kg)	70.44 (24.8)	70 (17.6)	0.679
Height(cm)	176.76(7.14)	175.96 (8.78)	0.724
VAS (0–10)	1.00 (1.00)	1.00 (1.00)	0.815

VAS: visual analog scale

### Comparison of clinical outcome measures within or between groups

Forty-six older adults completed the intervention (URT: *n* = 23; SRT: *n* = 23). Post-intervention outcomes are presented in Tables 3, 4 and 5.

**Table 3:** Baseline-adjusted between-group effects for outcomes meeting ANCOVA assumptions

Outcome		URT(*N* = 23)	SRT(*N* = 23)	Between-Groups Analysis of Covariance				
		Mean (SD)	Mean (SD)	Adjusted MD	95% CI	*P*	Holm-adjusted p	Effect size(ηp² )
BESS (0–60)	Pre	24.17 (6.34)	22.52 (5.99)	-4.22	-7.34 to -1.09	0.009	0.016	0.15
	Post	18.09 (3.52)	22.01 (6.56)					
	MD; 95% CI	-6.09; -9.25 to -2.92	-0.52; -3.59 to 2.56					
	*p*	0.001	0.731					
YBT right (%)	Pre	80.38 (13.56)	82.29 (10.77)	7.74	2.11 to 13.36	0.008	0.016	0.15
	Post	90.76 (8.85)	82.69 (10.21)					
	MD; 95% CI;	10.38; 2.44 to 18.31	0.40; -6.44 to 7.24					
	*p*	0.013	0.905					
YBT left (%)	Pre	81.98(11.46)	80.42 (11.23)	11.69	7.06 to 16.32	< 0.001	0.003	0.38
	Post	92.81(6.96)	81.11 (8.33)					
	MD; 95% CI;	10.82; 5.69 to 15.96	0.69; -5.88 to 7.25					
	*p*	< 0.001	0.830					
VL stiffness (m/s)	Pre	2.06 (0.47)	1.91 (0.29)	-0.56	-0.74 to -0.38	< 0.001	0.007	0.47
	Post	1.92 (0.34)	2.45 (0.28)					
	MD;95% CI;	-0.14; -0.34 to 0.06	0.53; 0.36 to 0.74					
	*p*	0.165	< 0.001					
SD co-activation	Pre	4.77 (0.42)	4.76 (0.24)	-0.53	-1.02 to -0.04	0.035	0.035	0.10
	Post	4.09 (0.91)	4.62 (0.72)					
	MD; 95% CI;	-0.68; -1.10 to -0.25	-0.14; -0.47 to 0.18					
	*p*	0.003	0.374					

Note: Baseline value was included as the covariate in the ANCOVA model. Adjusted MD represents the baseline-adjusted between-group mean difference estimated using ANCOVA. Holm-adjusted p values were used for multiple-comparison correction. Effect size is reported as partial eta squared (ηp²). *MD *mean difference, *CI *confidence interval, *BESS *Balance Error Scoring System, *YBT *Y-Balance Test, *VL *vastus lateralis, *SD *squat-down phase

**Table 4:** Between-group comparisons and baseline-to-week 12 changes

Variable	Stage	z	U	*p*-value #	HL estimate for Δ (95% CI)	Unadjusted *p* for Δ	Holm-adjusted *p* for Δ	Effect size *r*
Pain								
VAS (0–10)	Pre-test	-0.09	261.0	0.930	-1.00	0.009	0.009	0.39
	Post-test	-3.50	126.5	< 0.001	(-1.00 to 0.00)			
Quadriceps muscle stiffness (m/s)								
QT stiffness (m/s)	Pre-test	-0.60	237.0	0.546	0.07	0.560	0.924	0.09
	Post-test	-0.80	228.0	0.423	(-0.14 to 0.30)			
RF stiffness (m/s)	Pre-test	-1.17	211.5	0.244	-0.38	0.001	0.007	0.49
	Post-test	-3.14	121.5	0.002	(-0.61 to -0.16)			
VM stiffness (m/s)	Pre-test	-1.65	189.5	0.099	0.37	0.006	0.030	0.40
	Post-test	-4.35	66.5	< 0.001	(0.15 to 0.70)			
Hamstring muscle stiffness (m/s)								
HT stiffness (m/s)	Pre-test	-0.21	255.0	0.835	0.09	0.462	0.924	0.11
	Post-test	-1.15	212.0	0.249	(-0.16 to 0.38)			
BF stiffness (m/s)	Pre-test	-0.67	234.0	0.502	-0.34	0.018	0.072	0.35
	Post-test	-3.35	118.5	0.001	(-0.76 to -0.10)			
SM stiffness (m/s)	Pre-test	-1.70	187.0	0.089	-0.15	0.199	0.597	0.20
	Post-test	-1.59	192.0	0.111	(-0.42 to 0.12)			
Muscle co-activation								
SUSD co-activation	Pre-test	-0.25	253.0	0.801	-1.12	0.001	0.003	0.49
	Post-test	-3.09	124.0	0.002	(-1.96 to -0.79)			
SU co-activation	Pre-test	-1.79	183.0	0.073	-0.48	0.003	0.006	0.45
	Post-test	-2.45	153.0	0.014	(-0.95 to -0.19)			

Note: # = Mann–Whitney U test. VAS, visual analogue scale; QT, quadriceps tendon; RF, rectus femoris; VM, vastus medialis; HT, hamstring tendon; BF, biceps femoris; SM, semimembranosus; SU, stand-up phase; SUSD, transition phase between squat-down and stand-up. Δ was calculated as the post-intervention value minus the baseline value. The HL estimate represents the Hodges–Lehmann between-group median difference in change scores, calculated as ΔURT − ΔSRT. Unadjusted and Holm-adjusted p values refer to between-group comparisons of change scores from baseline to week 12. Effect size r was calculated as |Z|/√46

**Table 5:** Within-group changes from baseline to week 12

Group	URT group(*N* = 23)					SRT group(*N* = 23)			
Variable		Pre-test	Post-test	Z	*p*-value	Pre-test	Post-test	Z	*p*-value
Pain									
VAS (0–10)		1 (1)	0 (1)	-3.79	< 0.001	1 (1)	1 (0)	-2.71	0.007
Quadriceps muscle stiffness (m/s)									
QT stiffness (m/s)		2.35 (0.35)	2.59 (0.40)	-2.56	0.011	2.30 (0.31)	2.53 (0.33)	-2.25	0.024
RF stiffness (m/s)		1.97 (0.42)	1.99 (0.29)	-0.40	0.692	1.90 (0.33)	2.29 (0.19)	-3.77	< 0.001
VM stiffness (m/s)		1.71 (0.11)	2.18 (0.60)	-4.20	< 0.001	1.59 (0.34)	1.72 (0.29)	-0.99	0.323
Hamstring muscle stiffness (m/s)									
HT stiffness (m/s)		2.01 (0.37)	2.66 (0.54)	-4.20	< 0.001	1.97 (0.45)	2.52 (0.56)	-3.47	0.001
BF stiffness (m/s)		1.81 (0.07)	2.25 (0.67)	-1.80	0.073	1.79 (0.70)	2.44 (0.60)	-3.53	< 0.001
SM stiffness (m/s)		1.92 (0.19)	2.02 (0.56)	-0.54	0.592	2.04 (0.10)	2.16 (0.28)	-2.98	0.003
Muscle co-activation									
SUSD co-activation		4.87 (0.35)	2.45 (2.07)	-3.89	< 0.001	4.84 (0.20)	5.13 (1.94)	-1.28	0.201
SU co-activation		5.02 (0.52)	4.15 (1.02)	-4.11	< 0.001	4.90 (0.10)	4.51 (0.42)	-3.92	< 0.001

Note: Values are presented as median (IQR). *VAS *visual analog scale, *QT *quadriceps tendon, *RF *rectus femoris, *VM *vastus medialis, *HT *hamstring tendon, *BF *biceps femoris, *SM *semimembranosus, *SU *stand-up phase, *SUSD *transition phase between squat-down and stand-up

#### Muscle stiffness

Between-group comparisons of changes from baseline to week 12 showed greater increases in stiffness after SRT than after URT for RF (*p* = 0.001), BF (*p* = 0.018), and VL (*p* < 0.001). In contrast, VM showed a greater increase after URT than after SRT (*p* = 0.006) (Table 4). After Holm adjustment, only RF, VL, and VM remained significant, whereas BF, QT, HT, and SM were non-significant (Tables 3 and 4). Within-group analyses showed significant increases in QT (*p* = 0.011), VM (*p* < 0.001), and HT (*p* < 0.001) after URT, and significant increases in QT, HT, VL, RF, BF, and SM after SRT (all *p* < 0.05) (Table 5).

#### Muscle co-activation

Between-group comparisons of changes from baseline to week 12 indicated greater reductions in co-activation after URT than after SRT during SUSD (Holm-adjusted *p* = 0.003), SU (Holm-adjusted *p* = 0.006), and SD (Holm-adjusted *p* = 0.035) (Tables 3 and 4). The URT group showed significant within-group reductions in co-activation during SUSD (*p* < 0.001), SU (*p* < 0.001), and SD (*p* = 0.003). The SRT group showed no significant within-group reductions except during SU (*p* < 0.001) (Table 5).

#### Balance performance

Baseline-adjusted between-group comparisons revealed greater improvements after URT than after SRT in BESS (Holm-adjusted *p* = 0.016), YBT-right (Holm-adjusted *p* = 0.016), and YBT-left (Holm-adjusted *p* = 0.003) (Table 3). The URT group exhibited significant within-group improvements in static balance, as measured by BESS (*p* = 0.001), and in dynamic balance, as assessed by YBT-right (*p* = 0.013) and YBT-left (*p* < 0.001). The SRT group did not demonstrate significant within-group changes for BESS (*p* = 0.731), YBT-right (*p* = 0.905), or YBT-left (*p* = 0.830) (Table 5).

#### Knee Pain Intensity

Between-group comparison of changes from baseline to week 12 indicated a greater reduction in VAS in the URT group than in the SRT group (*p* = 0.009) (Table 4). The result remained significant after Holm adjustment. After the 12-week intervention, both groups showed significant within-group reductions in VAS (Table 5).

## Discussion

### Key Findings and context

This randomized trial is the first to directly compare unstable resistance training (URT) versus stable resistance training (SRT) on muscle stiffness, co-activation, balance, and pain in older adults. Both modalities produced significant reductions in pain intensity, suggesting that resistance training may provide pain-related benefits for older adults. However, the between-group findings showed that URT led to greater neuromuscular efficiency, as shown by significantly reduced co-activation across SD, SUSD, and SU (*p* < 0.05), as well as improvements in static balance and dynamic balance (all *p* < 0.05). Notably, a novel finding was that URT’s stiffness adaptations were muscle-specific—limited to the quadriceps tendon (QT), hamstring tendon (HT), and vastus medialis (VM) (all *p* < 0.05)—whereas SRT produced broader stiffness gains across muscle groups. This suggests that integrating instability-based training into resistance programs may help target age-related neuromuscular and balance challenges. Specifically, this combined approach may optimize co-activation and improve balance, both of which are relevant to fall-prevention mechanisms and functional mobility in older adults [44, 45].

### Muscle-specific stiffness adaptations with URT

To our knowledge, this is the first study showing that URT improves muscle stiffness in a muscle-specific, not generalized, manner, with effects limited to the QT, HT, and VM. These muscles are key knee stabilizers and are highly engaged during repetitive loading and postural demands, likely to explain their selective adaptation. In contrast, SRT induces broader quadriceps and hamstring stiffness changes through greater mechanical loading and general strengthening. These differences suggest distinct adaptation pathways: URT increases stabilizer stiffness by inducing neural adaptations via balance-driven sensorimotor challenges [46]. Initial URT-induced instability may temporarily reduce joint tension control by taxing sensorimotor resources. With repeated training, the instability raises spinal reflex sensitivity and α-motor neuron excitability [47, 48]. This process improves sensorimotor integration, enabling the nervous system to adjust tension and stiffness more efficiently [47, 49].

Furthermore, unlike SRT, which relies on high mechanical loads, URT boosts active stiffness through neuromuscular regulation even at moderate loads [50, 51]. This aligns with evidence that moderate-load training (e.g., 55% 1RM) can enhance tendon mechanical properties in older adults [52, 53], highlighting the role of neural control over load magnitude in stiffness adaptation. Additionally, our study found that URT reduced maladaptive co-activation; this adaptation may support greater muscle–tendon stiffness by lowering tendon strain and refining muscle mechanics [54–56]. These findings highlight URT’s value as a low-load, high-function approach for improving muscle stiffness in older adults, with less risk than high-load training.

### Phase-specific co-activation modulation with URT

In this study, URT significantly reduced muscle co-activation across SU, SD, and SUSD (all *p* < 0.05). In contrast, SRT showed improvement only during SU, with no significant changes in the other phases (*p* > 0.05). These findings indicate superior neuromuscular coordination and efficiency with URT in older adults. Unlike SRT, which emphasizes linear, externally loaded movements with limited proprioceptive input [57], URT introduces balance perturbations that increase sensorimotor integration and postural control demands across the kinetic chain [57, 58]. A key adaptation is improved agonist–antagonist coordination, which likely reduces co-activation by promoting more efficient reciprocal inhibition and motor unit recruitment [59].

Recent EEG-based evidence shows that instability alters cortical activation in key motor areas during bodyweight squats, promoting optimized intermuscular coordination and reduced redundant muscle activity [60].This neuromuscular refinement is particularly important for older adults, who often compensate for reduced proprioception and motor control by increasing superficial muscle co-activation. Our observation of reduced co-activation during the transition phase (SUSD) suggests this compensation is suboptimal and indicates that URT may address it by promoting activation of deeper trunk and hip stabilizers [61, 62]. Although previous studies have indicated that resistance training maintains or increases co-activation under high-load or unstable conditions [63], the present URT findings demonstrated a reduction in co-activation. This decrease may reflect enhanced neuromuscular efficiency and diminished compensatory activity, aligning with outcomes reported for hybrid and sensorimotor-based resistance interventions [57, 64]. These results further support the potential relevance of URT for improving neuromuscular coordination.

### Greater dynamic than static balance improvements with URT

The greater improvements in both dynamic and static balance compared with SRT may result from URT’s enhanced proprioceptive acuity and neuromuscular responsiveness [65]. Repeated postural perturbations during URT engage proprioceptive feedback and spinal reflexes, fostering faster sensorimotor integration and adaptive muscle responses [66, 67]. This can improve coordination among lower-limb synergists and stabilizers, likely helping joint alignment and movement accuracy during balance tasks [65]. However, Eckardt [23] found no clear difference between URT and SRT in improving balance among older adults, possibly due to low instability intensity and limited perturbation variability in their study. Specifically, a “semi-unstable” environment (e.g., stable equipment with minor unstable elements) increases balance demand but does not create enough sensorimotor challenge to drive substantial vestibulo–spinal–cortical adaptation [59]. Also, fixed and predictable instability might result in early neural habituation, which could limit long-term sensorimotor gains [68]. In our study, URT progressively increased instability with a non-fixed platform, providing sustained, unpredictable balance challenges that likely increased sensorimotor engagement and adaptation.

Interestingly, URT produced greater gains in dynamic balance (YBT scores: right leg: MD = 10.38 or left leg MD = 10.82, vs. BESS: MD=-6.09) compared to static balance in our study. The superior dynamic balance likely arises because URT specifically targets sensorimotor adaptability, requiring continuous neuromuscular coordination in response to unpredictable external mechanical perturbations. In contrast, static balance relies primarily on intrinsic viscoelastic and skeletal postural stabilization mechanisms, which do not demand the same level of sensorimotor adjustment. These findings align with the mechanisms discussed above, but other studies report differing results. Martínez-Amat et al. [69] found that their proprioceptive training utilized Swiss balls and BOSU tools produced larger improvements in static balance than in dynamic balance. Differences in training approaches likely explain this: their intervention involved instability devices but did not incorporate progressively increasing loads or unexpected challenges, resulting in a less pronounced sensorimotor challenge. Furthermore, URT reduced excessive co-activation in the present study; however, balance gains persisted beyond this effect [70]. This may imply that URT elicited additional adaptations related to passive joint stabilization, which may have contributed to improved balance performance [71, 72]. However, actual falls were not assessed; therefore, the balance findings should not be interpreted as direct evidence of reduced fall risk.

### Greater VAS reduction with URT

Although pain perception was not the primary focus of this study, significant improvements in VAS scores were observed in both groups (both *p* < 0.05) as additional findings. Furthermore, between-group comparison of changes from baseline to week 12 showed a greater reduction in VAS with URT than with SRT (*p* = 0.009). These findings are partially consistent with those of Eckardt et al. [26], who reported that instability resistance training led to greater improvements in cognitive and motor functions compared to stable resistance training in healthy older adults. While their study primarily investigated executive function rather than pain perception, the neuromuscular adaptations associated with URT may help reduce discomfort, aligning with the VAS improvements observed in the present study.

### Implications

Our findings offer valuable guidance for designing targeted exercise interventions for older adults. Progressive URT is feasible for this population and can produce potentially beneficial neuromuscular and balance-related adaptations. These include greater muscle–tendon stiffness in VM, QT, and HT, reduced maladaptive co-activation, and improved static and dynamic balance, all with moderate loads. Compared with SRT, URT appears to better foster sensorimotor adaptability and co-activation regulation, whereas SRT may be more suitable for broader stiffness-related adaptations. Programs may consider adding controlled instability to resistance sessions to enhance proprioception, reflex responsiveness, and intermuscular coordination, thereby potentially improving joint control and balance. Future work should define dose–response and long-term effects, and determine if these improvements extend to daily function, fall prevention, and sustained independence.

### Limitations and future directions

Future studies should address the limitations of the current study by increasing sample size and broadening eligibility criteria to enhance generalizability across older adult subgroups. Incorporating a non-training control group or dose-equated comparators would allow for clearer isolation of modality effects from total training dose. Extending follow-up to 6–12 months is needed to assess the durability of changes in stiffness, co-activation, balance, and pain. Additionally, actual falls, functional independence, and prespecified MCID thresholds for pain or balance outcomes were not assessed, which limits the interpretation of clinical relevance.

## Conclusions

The study highlights the benefits of URT, which produced selective stiffness adaptations, reduced co-activation, and improved balance among older individuals. This comprehensive training approach has the potential to support neuromuscular and balance-related adaptations in the elderly. Future research should explore the long-term effects of combined interventions and examine their applicability across different populations. Fall and joint prevention programs may consider URT as a complementary strategy, but further studies are needed to determine if laboratory improvements translate into fewer falls and greater functional independence in real-world scenarios.

## Supplementary Information

Supplementary Material 1.

## Data Availability

No datasets were generated or analysed during the current study.

## Appendix material

### Appendix 1: Muscle stiffness measurement (Supplementary Appendix 1)

Participants wore shorts to allow full exposure of the thigh region and removed their shoes to avoid interference during imaging. A generous layer of ultrasound gel was applied to minimize acoustic distortion, and care was taken to avoid exerting pressure with the probe. During stiffness measurements, the ultrasound probe was positioned parallel to the orientation of the muscle fibres to ensure consistent image quality and measurement accuracy [73].To ensure image consistency, the muscle was stratified into upper, middle, and lower layers, with the elastography imaging field fixed in the middle layer [74, 75]. The region of interest (ROI) was standardized to 1 × 1 mm. After stabilizing the elastography sampling box (a square imaging field automatically generated by the VTIQ mode) for 5 s, the image was frozen. Within this box, ROI was positioned in the middle layer of the muscle to obtain SWV values. For each muscle, three images were captured, and three ROIs were selected per image. The average of all measurements was used for subsequent analyses.

### Appendix 2 (Supplementary Appendix 2)

#### (a)MVIC test

MVIC is commonly used to normalize electromyographic (EMG) data for standardized comparisons across participants and conditions. Quadriceps and hamstrings MVICs were measured bilaterally using standardized resistance procedures [76, 77]. MVIC testing was performed twice for each muscle group, with a 1-minute rest between trials, in seated and prone positions. Raw EMG signals were sampled at 2000 Hz, bandpass-filtered (20–460 Hz), and amplified (CMRR 130 dB, input impedance 20,000 kΩ). Then, the EMG activity was processed by calculating the root mean square (RMS), with the highest EMG_RMS_ values from the two trials extracted and averaged for subsequent data normalization. During the testing, participants were required to remain barefoot and wear loose-fitting shorts to allow full visibility of the thigh region.

#### (b)MVIC measurement

Quadriceps MVIC Measurement: Participants were seated upright in a chair with their hips and knees at 60° and 30° of flexion. The experimenter applied manual resistance to the distal end of the calf, and participants were instructed to extend their knees with maximum effort against the resistance. Each MVIC trial lasted 3–5 s, with sufficient rest periods to prevent fatigue.

Hamstring MVIC Measurement: Participants lay prone on a treatment bed with the knee joint positioned at 60° of flexion. The experimenter applied resistance to the distal end of the calf, instructing participants to flex their knees with maximum effort. Each MVIC trial was maintained for 3–5 s, with adequate rest intervals to prevent fatigue.

### Appendix 3: Muscle co-activation measurement (Supplementary Appendix 3)

Muscle co-activation refers to the simultaneous activation of agonist and antagonist muscle groups, such as the quadriceps and hamstrings, to enhance joint stability and movement control; excessive co-activation can lead to increased joint stiffness and reduced mobility [14, 15]. Muscle co-activation index was calculated using the time-varying multi-muscle co-activation function (TMCf) and the time-varying multi-muscle co-activation index (TMCi) [37]. TMCi quantifies the degree of simultaneous activation between agonist and antagonist muscles over time, expressed as a percentage (%) of total activation. Higher TMCi values indicate greater muscle co-activation, reflecting increased neuromuscular effort.

$$\begin{array}{l}TMCF\:(d(t),t)=(1-\frac{1}{1+\:e^{-12(d(k)-0.5)}}).\frac{(\:{\sum\:}_{{i}{=1}}^{{N}}{EMGi}\mathrm{(}\mathrm{t}{)/({100}\:\times\:{N}))^{2}}}{max_{i=1{\dots\dots{N^{[EMGi(t)]}{}}}}}\end{array}$$

$$\:{TMCi}\mathrm{=}\frac{{\sum\:}_{\mathrm{t}\mathrm{=1}}^{\mathrm{100}}{TMCf}{(}{t}\mathrm{)}}{\mathrm{100}}$$

Surface landmarks for RF, VL, VM, ST, and BF were identified in accordance with established guidelines [35]. EMG data were collected at 2000 Hz. Subjects performed specific movements post-electrode placement within an optimal signal capture area. Raw EMG signals were amplified using a differential amplifier (CMRR 130dB, input impedance 20,000 kΩ) and band-pass filtered (20 Hz to 460 Hz) with a Butterworth filter. The filtered EMG data related to each trial were expressed as a percentage of the EMG peak value calculated as the mean of the maximum values detected for each of the two MVICs. All five acquired muscles were inserted in the calculation of the TMCf to assess co-activation.

The selection of squatting and standing tasks is biomechanically and clinically justified, as they effectively assess lower limb function, joint stability, and neuromuscular adaptation [78, 79]. Squat-down task (SD): Subjects performed squats from shoulder-width apart, toe-forward position to maximum depth. Stand-up task (SU): Subjects rose from squatting to fully standing. Squat-down to stand-up task (SUSD): Subjects continuously performed the full motion from the initial squatting position to full standing, incorporating eccentric and concentric muscle actions. To minimize variability caused by transitional movements, the first and fifth squatting and standing trials under each condition were excluded. For each participant, data from the remaining three trials per condition were averaged for analysis. Within each phase (SD, SU, and SUSD), the synthetic co-activation index wascalculated as the mean value of the TMCf curve, representing the average co-activation level throughout the movement cycle (% co-activation). All algorithms were implemented using MATLAB software (version 2018b; MathWorks, Natick, MA, USA).
